# Values, intercultural sensitivity, and uncertainty management: a cross-cultural investigation of motivational profiles

**DOI:** 10.3389/fpsyg.2025.1623929

**Published:** 2025-08-06

**Authors:** Jungsun Kim, Huai-Rhin Kim

**Affiliations:** ^1^Center of Religion and the Global East, Purdue University, West Lafayette, IN, United States; ^2^School of Languages and Cultures, Purdue University, West Lafayette, IN, United States

**Keywords:** personal values, latent profile analysis, intercultural sensitivity, intolerance of uncertainty, cross-cultural comparison, university students, value integration, motivational profiles integrative traditionalists

## Abstract

**Introduction:**

Personal values function as core motivational forces shaping cognition and behavior. However, the interaction between these values, intercultural sensitivity, and intolerance of uncertainty across cultures has received limited empirical attention. This study investigates how these constructs combine to form distinct motivational profiles among university students in South Korea and the United States.

**Methods:**

Using a person-centered latent profile analysis (LPA), we identified value configurations among South Korean (N = 517) and U.S. (N = 431) undergraduates. Participants completed the Portrait Values Questionnaire–Revised Revised (PVQ-RR), Intercultural Sensitivity Scale (ISS), and Intolerance of Uncertainty Scale–Short Form (IUS-12). Multinomial logistic regression identified predictors of profile membership.

**Results:**

Among Korean students, five value profiles emerged: Integrative Traditionalists (41%), Low Tradition Endorsement (24.2%), Change-Oriented (21.1%), Low Tradition/High Openness (17.5%), and Tradition-Oriented (21.8%). The U.S. sample revealed four profiles: Growth-Oriented (24.8%), Broad Value Endorsement (21.8%), Security-Focused (35.0%), and Low Tradition Endorsement (18.3%). Across both samples, higher intercultural engagement and confidence predicted membership in growth-oriented profiles, while elevated inhibitory intolerance of uncertainty and lower engagement characterized tradition- or security-focused profiles. Gender effects appeared only in Korea, where women demonstrated greater likelihood of belonging to tradition-oriented groups.

**Discussion:**

These findings challenge assumptions about the incompatibility between tradition and openness values in Schwartz’s framework. Cultural tightness and individual psychological dispositions appear to jointly shape motivational value integration. The results have implications for designing culturally responsive interventions that enhance intercultural competence and promote adaptive value systems in diverse educational settings.

## 1 Introduction

The quest to understand human values lies at the heart of psychological, sociological, and intercultural research. Values function as powerful motivational structures that shape cognition and behavior (Schwartz, 2012), guide ethical decision-making processes (Mellers et al., 1998), and establish belief frameworks that direct human action (Verplanken and Holland, 2002). Among theoretical frameworks, Schwartz’s theory of basic human values stands as preeminent, identifying a universal structure of motivational goals that transcend cultural boundaries (Schwartz and Bilsky, 1987; Schwartz, 2012). This model has evolved from its original 10 value types to a more nuanced framework of 19 values, arranged in a circular structure that elegantly captures their complementary relationships and inherent tensions. This circular arrangement facilitates sophisticated cross-cultural comparisons while accommodating both individualistic and collectivistic orientations.

Values extend beyond personal decision-making to fundamentally influence how individuals navigate cultural diversity. Intercultural sensitivity—the capacity to recognize, respect, and appropriately respond to cultural differences—demonstrates consistent associations with particular value orientations (Chen, 1997). For instance, individuals prioritizing openness to change or universalism typically embrace cultural differences more readily, while those emphasizing conservation or security often approach intercultural encounters with greater reservation. While intercultural sensitivity develops through experiential learning and reflection, it remains profoundly influenced by value-based predispositions that color affective, behavioral, and cognitive dimensions of cross-cultural communication.

Recent research suggests that psychological dispositions, particularly Intolerance of Uncertainty (IU), serve as critical mediating factors in this value-sensitivity relationship. IU represents a dispositional difficulty in tolerating ambiguity, unpredictability, or incomplete information—conditions inherent to intercultural exchanges (Freeston et al., 1994; Dugas et al., 1998; Tanovic et al., 2018). Individuals with elevated IU typically perceive uncertain situations as threatening, potentially compromising their willingness to engage with unfamiliar cultural norms. In our increasingly complex global society—characterized by pandemic disruptions, accelerating digital transformation, and volatile geopolitical landscapes—understanding the interplay between values, IU, and intercultural sensitivity has acquired unprecedented theoretical and practical significance.

Schwartz and Bilsky (1990) emphasized that value hierarchies exhibit distinct cultural patterns, underscoring the necessity of cross-national investigations to illuminate how values manifest across diverse sociocultural contexts. Our study examines undergraduate students from South Korea and the United States—representing contrasting Eastern and Western paradigms—to explore how distinct value profiles relate to both intercultural sensitivity and tolerance for uncertainty. By employing latent profile analysis (LPA), a sophisticated person-centered approach, we aim to uncover not merely the architecture of individual value profiles but also how psychological and intercultural dispositions predict profile membership.

This investigation addresses two central research questions:

1. What distinctive value profiles emerge among undergraduate students in Korea and the United States?
2. How do intercultural sensitivity and intolerance of uncertainty predict membership across these value profiles?

We advance two primary hypotheses:

*H1:* Culturally distinct value profiles will emerge within each national sample, reflecting culturally shaped patterns of value prioritization.

*H2:* Intercultural sensitivity and intolerance of uncertainty will significantly predict value profile membership, with culturally distinctive association patterns between the two countries.

In addition, this study aims to explore how the structure and prevalence of value profiles may reflect theoretical differences in cultural tightness-looseness and collectivism-individualism. This comparative perspective is grounded in Gelfand et al.’s (2011) tightness-looseness framework and prior research on Confucian and Western value systems.

Accordingly, we conceptually hypothesize that IU may mediate the relationship between personal values and ISS, and that cultural tightness–looseness may moderate these pathways. While the present study employs LPA to identify latent profiles, these theoretical links provide a basis for future research using SEM and longitudinal designs.

**Figure 1:**
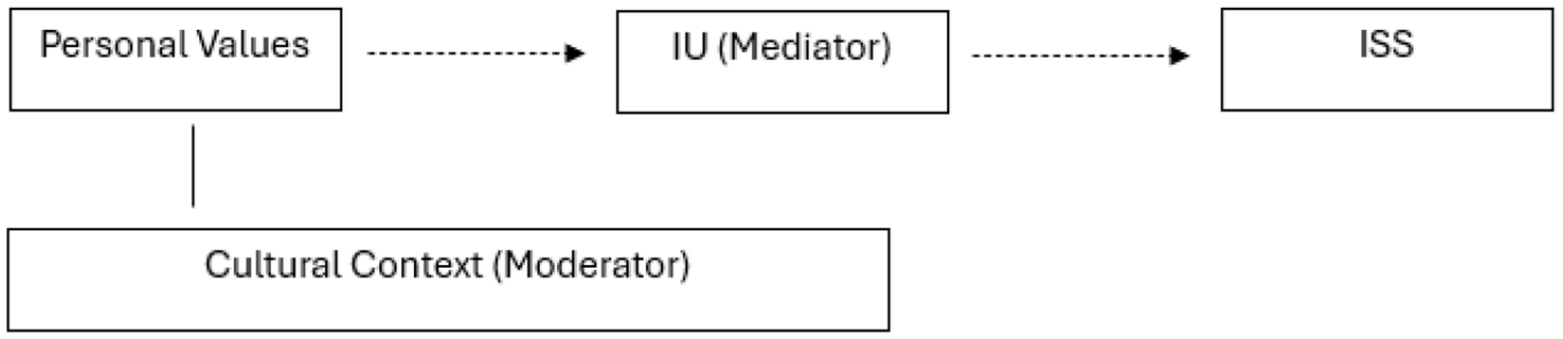
Conceptual model linking values, sensitivity, and uncertainty.

To visually clarify the conceptual pathways implied by our framework, Figure 1 illustrates the proposed relationships among personal values, intolerance of uncertainty (IU), and intercultural sensitivity (ISS). In this hypothetical model, IU is positioned as a potential mediator linking personal values to ISS, while cultural context—operationalized as tightness–looseness—may moderate the pathway from personal values to IU. This conceptualization reflects theoretical assumptions drawn from tightness–looseness theory (Gelfand et al., 2011) and prior work on value rigidity and openness.

## 2 Materials and methods

### 2.1 Literature review

#### 2.1.1 Schwartz’s value theory

Schwartz’s theory of basic human values offers a comprehensive framework for understanding what motivates people across different cultures. Initially featuring 10 broad value types, the model has evolved to include 19 distinct values arranged in a circular pattern that shows how they relate to each other (Schwartz et al., 2012). This circular arrangement is not random—it reflects that adjacent values have compatible motivations, while opposing values represent conflicting goals. The refined model also distinguishes values along dimensions like self-protection versus growth and personal versus social focus, giving us a deeper understanding of how value priorities shape and are shaped by our experiences.

Research has confirmed that this circular structure of values is recognized across many different societies, even though specific values may be prioritized differently depending on the culture. For instance, Sortheix and Schwartz (2017) found that values emphasizing openness to change (which focus on growth and personal development) tend to be linked with greater subjective well-being, while conservation values (which emphasize self-protection and social focus) often show negative correlations with well-being. These findings highlight how values not only guide individual behavior but also influence broader outcomes like happiness and economic performance.

Despite its wide applicability, comparing values across cultures remains challenging. Recent work by He et al. (2017) compared different methods for making value measurements more comparable across countries. While no single approach solves all issues of measurement consistency, techniques like anchoring vignettes and treating data as ordered categories can help improve comparability. Researchers need to apply these methods carefully, staying aware of potential biases from response styles or translation differences—especially important in cross-national studies like ours that examines patterns in value priorities among Korean and U. S. undergraduate students.

In summary, Schwartz’s refined value theory provides a robust, well-tested framework for examining how values interact within and across cultures. Its circular structure, multidimensional distinctions, and methodological rigor make it an essential foundation for contemporary research on values, well-being, and intercultural adaptation.

#### 2.1.2 Intercultural sensitivity

Intercultural sensitivity forms the emotional foundation of intercultural competence, reflecting a person’s ability to recognize, respect, and appropriately respond to cultural differences (Chen and Hu, 2023). Conceived as the affective dimension of intercultural communication competence, it enables people to engage effectively and appropriately across cultures. This emotional orientation is considered essential for successful cross-cultural interactions, helping individuals recognize differences in multicultural environments, respect them, and communicate effectively.

Researchers understand intercultural sensitivity as multidimensional, involving emotional, cognitive, and behavioral elements. Bennett (1986, 1993) described it as a developmental journey where people move from ethnocentric to ethno-relative worldviews as they become more aware and accept differences. Hammer et al. (2003) clarified that while intercultural sensitivity refers to the internal, psychological ability to perceive and experience cultural differences, intercultural competence concerns the external behaviors shown in cross-cultural situations. As people develop greater sensitivity, their competence becomes more natural and less dependent on following prescribed rules, reflecting a deeper understanding of cultural diversity.

The Intercultural Sensitivity Scale (ISS) developed by Chen and Starosta (2000) has become widely used because of its strong measurement properties. The ISS includes 24 items across five areas: interaction engagement, respect for cultural differences, interaction confidence, interaction enjoyment, and interaction attentiveness. The scale has shown high reliability (Cronbach’s *α* = 0.88) and has been validated across diverse populations in countries including China, Korea, Malaysia, Germany, the United States, and Chile. Studies using the ISS have enabled detailed comparisons of intercultural sensitivity across groups and cultures, as well as investigations into what influences sensitivity and how it relates to communication competence.

Recent research emphasizes that intercultural sensitivity is not a fixed trait but a skill that develops through direct experience, education, and reflection. For example, exposure to multicultural environments and participation in intercultural workshops have been shown to enhance students’ sensitivity, particularly in emotional and behavioral aspects (Ichikawa and Kim, 2025). However, cross-cultural comparisons reveal that the expression and development of intercultural sensitivity are shaped by local context. In Korea, for instance, researchers have documented students’ transition from a traditionally homogeneous outlook to a more heterogeneous perspective, with unique emphasis on overcoming social prejudices and actively engaging with cultural differences. Despite this progress, defensive patterns—such as avoiding or withdrawing from intercultural conflict—remain common, highlighting the need for contextually sensitive approaches to developing intercultural sensitivity.

In summary, intercultural sensitivity is a multifaceted, developmental quality that underlies effective intercultural communication. While established measurement tools like the ISS facilitate cross-cultural research, ongoing studies continue to refine our understanding of how intercultural sensitivity is shaped by individual values, psychological dispositions, and sociocultural context (Chen and Starosta, 2000).

#### 2.1.3 Intolerance of uncertainty

Intolerance of Uncertainty (IU) describes a person’s difficulty in handling the discomfort that comes from not having enough information in ambiguous situations. Those with high IU tend to see uncertainty as threatening (Morse et al., 2021). People who struggle with uncertainty often seek information and closure to restore a sense of predictability and control when facing unclear situations. This tendency shows up as a stronger need for cognitive closure, preference for order, and sometimes closed-mindedness, which can lead to rushing decisions rather than exploring multiple possibilities. When uncertainty cannot be resolved, those with high IU typically experience increased anxiety and worry, often leading them to avoid situations and experience psychological distress.

Recent research views IU as having two related dimensions: prospective IU and inhibitory IU. Prospective IU involves the desire for predictability and the tendency to assess potential threats related to future uncertainty, often resulting in active information-seeking behaviors. In contrast, inhibitory IU reflects feeling paralyzed or avoiding situations when facing uncertainty, such as being unable to act when outcomes aren’t clear. While these dimensions represent different aspects of IU, they are strongly connected and may function differently depending on the context: those high in prospective IU might try to resolve uncertainty through action, while those high in inhibitory IU might withdraw from uncertain situations altogether.

The IU is particularly relevant for university students, who navigate numerous uncertainties in their academic, career, and social lives. Higher levels of IU have been linked to greater psychological distress, including depression and anxiety, especially during highly uncertain periods like the COVID-19 pandemic (Zhang et al., 2024). A recent study of Chinese college students found that IU was significantly associated with depressive symptoms, and that coping strategies played an important mediating role: students with high IU who relied on negative coping strategies were more likely to report depression, while those using positive coping strategies showed more resilience. These findings highlight the importance of developing adaptive coping skills and tolerance for uncertainty to protect student mental health.

In intercultural contexts, IU plays a critical role in shaping attitudes and behaviors toward cultural differences. People with high IU are more likely to see unfamiliar cultural norms as threatening, which can lead to avoidance or controlling approaches in intercultural interactions (Syrtsova, 2014). Studies of international students have found that higher IU scores correlate with greater use of avoidance and dominating strategies, and lower preference for integrative or compromising approaches to conflict. This suggests that IU not only contributes to psychological distress but also influences how individuals handle intercultural challenges, potentially hindering effective communication and adaptation.

In summary, IU is a multidimensional construct that significantly influences both psychological well-being and intercultural engagement. Its role as a mediator between values and intercultural sensitivity highlights the need for interventions that help students become more comfortable with uncertainty and develop adaptive coping strategies, particularly in increasingly diverse and unpredictable environments.

#### 2.1.4 Conceptual integration and gaps

To clarify the theoretical pathways implied by our conceptual framework, we posit that intolerance of uncertainty (IU) may function as an intermediary psychological mechanism linking personal values to intercultural sensitivity (ISS). Specifically, individuals’ value orientations may shape their tolerance for uncertainty, which in turn influences their openness to diverse cultural contexts. Furthermore, cultural context—conceptualized as tightness–looseness (Gelfand et al., 2011)—may moderate these pathways, such that the relationships among values, IU, and ISS differ systematically across cultural settings. This conceptual model highlights the need for future research to test these directional pathways using mediation and moderation analysis, which goes beyond the scope of the present person-centered LPA approach. The interplay among values, intercultural sensitivity, and intolerance of uncertainty (IU) represents a crucial intersection for understanding how people navigate increasingly complex, multicultural environments. Our study integrates these concepts using a person-centered approach through latent profile analysis (LPA), moving beyond traditional variable-centered methods to capture nuanced patterns of value prioritization and their psychological and intercultural correlates.

Recent research shows that value orientations, as described by Schwartz’s refined theory, are not only shaped by culture but also dynamically interact with psychological dispositions and intercultural competencies. For example, people who prioritize openness to change and universalism tend to show higher levels of intercultural sensitivity, reflecting a greater willingness to engage with and appreciate cultural differences. On the other hand, those with stronger conservation or security values may approach intercultural situations more cautiously, often influenced by their underlying tolerance or intolerance for uncertainty.

Intolerance of uncertainty emerges as a key mediator in this relationship. People with high IU are more likely to perceive ambiguous or unfamiliar cultural encounters as threatening, which can reduce their intercultural sensitivity and lead to less adaptive conflict management strategies, such as avoidance or dominance (Syrtsova, 2014). This dynamic is particularly relevant for university students, who face both developmental and situational uncertainties in increasingly diverse educational settings. Integrating these constructs allows for a more holistic understanding of how value profiles, psychological dispositions, and intercultural skills come together to shape cross-cultural adaptation.

The application of LPA in this study enables us to identify distinct value profiles and their associations with intercultural sensitivity and IU. This approach aligns with recent work in healthcare education, where LPA has revealed diverse profiles of intercultural sensitivity—such as “interculturally sensitive,” “interculturally uncertain,” and “interculturally refusing”—each with unique predictors and needs (Lucza et al., 2024). Such findings highlight the importance of tailored interventions that address not only value orientations but also psychological vulnerabilities and strengths.

Despite these advances, several gaps remain:

- Limited Integration Across Constructs: Few studies have simultaneously examined value profiles, intercultural sensitivity, and IU within a unified, person-centered analytic framework, especially in non-Western contexts.
- Cultural Specificity: There’s a shortage of research exploring how these relationships manifest differently across cultures. For instance, the role of tradition and face in East Asian value systems, or the impact of anxiety-related value dimensions in Western samples, remains underexplored.
- Mechanisms of Influence: The mediating or moderating role of IU in the link between values and intercultural sensitivity is not well understood, particularly regarding how IU may amplify or buffer the effects of certain value orientations on intercultural engagement and adaptation.
- Developmental and Educational Implications: While LPA studies in educational settings have begun to identify vulnerable subgroups (e.g., “interculturally uncertain” students with high empathy but low confidence), more research is needed on effective interventions that can strengthen intercultural sensitivity and reduce IU in these populations (Lucza et al., 2024).

By integrating value theory, intercultural sensitivity, and IU through a person-centered lens, this study addresses critical gaps in the literature and provides a foundation for more nuanced, culturally responsive research and practice. Future work should further explore the mechanisms linking these constructs and develop targeted interventions to foster adaptive value profiles and intercultural competencies in diverse educational and organizational contexts (Lucza et al., 2024; Syrtsova, 2014).

### 2.2 Method

#### 2.2.1 Research design

To examine the underlying structure of human values and their associations with intercultural sensitivity and intolerance of uncertainty (IU) across cultural contexts, this study employed a person-centered approach using Latent Profile Analysis (LPA). Unlike traditional variable-centered approaches that simply compare average value scores between demographic groups, LPA helps us identify distinct subgroups of individuals who share similar patterns of values. This approach is particularly valuable for understanding the complex relationships between motivational value structures, attitudes toward other cultures, and psychological characteristics across culturally diverse populations.

#### 2.2.2 Participants

A total of 948 undergraduate students participated: 517 from South Korea and 431 from the United States. Key demographic differences are summarized in Table 1.

**Table 1:** Participants demographic differences.

Characteristic	Korean sample	U. S. sample
Recruitment source	Multiple universities via survey company	Single research university (STEM-focused)
Age (*M* ± SD)	22.12 ± 1.87	20.56 ± 1.45
Gender	250 male, 267 female	165 male, 266 female
Ethnicity	100% Korean	56.6% White, 30.9% Asian, 8.1% Black, 4.4% Other

#### 2.2.3 Measures

Participants completed the Portrait Values Questionnaire-Revised Revised (PVQ-RR; Schwartz et al., 2012), which measures 19 distinct personal values. For each item, participants indicated on a 6-point Likert scale how similar they felt to a fictional character described in the statement. For this study, we focused on 12 values representing the 10 original value types plus two culturally significant additions—humility and face. The Korean version was validated by Choi and Lee (2014) and reviewed by Schwartz himself. Reliability coefficients (Cronbach’s alpha) ranged from 0.54 to 0.87 across the 12 values in the Korean sample.

The 24-item Intercultural Sensitivity Scale (ISS; Chen and Starosta, 2000) was used to assess five dimensions: Interaction Engagement, Respect for Cultural Differences, Interaction Confidence, Interaction Enjoyment, and Interaction Attentiveness. Items were rated on a 5-point Likert scale (1 = strongly disagree to 5 = strongly agree). The ISS has demonstrated strong reliability and cross-cultural validity in multiple international samples (Table 2).

**Table 2:** Measurement constructs, instruments, subscales, and reliability estimates.

Construct	Instrument	Subscales	Sample item	Cronbach’s α (KR/US)
Human values	PVQ-RR	12 values (e.g., Tradition, Universalism)	“It is important to him/her to follow traditions.”	0.81/0.79
Intercultural sensitivity	ISS	Engagement, Respect, Confidence, Enjoyment, Attentiveness	“I enjoy interacting with people from different cultures.”	0.76/0.83
Intolerance of uncertainty	IUS-12	Prospective IU, Inhibitory IU	“Uncertainty makes me uneasy.”	0.87/0.85

KR, Korean sample; US, United States sample. PVQ-RR, Portrait Values Questionnaire–Revised Revised (Schwartz et al., 2012); ISS, Intercultural Sensitivity Scale (Chen and Starosta, 2000); IUS, Intolerance of Uncertainty Scale–Short Form (Carleton et al., 2007).

To measure intolerance of uncertainty, we used the 12-item Intolerance of Uncertainty Scale–Short Form (IUS-12; Carleton et al., 2007). This scale includes two subscales: Prospective IU (desire for predictability and anticipatory worry) and Inhibitory IU (paralysis when facing uncertainty). Participants rated items on a 5-point Likert scale (1 = not at all characteristics of me to 5 = entirely characteristic of me).

#### 2.2.4 Procedure

Participants in both countries completed the surveys online. The Korean version of the questionnaire underwent rigorous translation and back-translation procedures, ensuring linguistic and conceptual equivalence across languages. Institutional Review Board (IRB) approval was obtained from the authors’ institutions, and all participants provided informed consent after receiving detailed study information. The survey platform incorporated mechanisms to prevent duplicate responses and included attention-check items to maintain data integrity. Participation was restricted to eligible undergraduate students, with responses that failed quality assessments systematically excluded from analysis. All procedures received Institutional Review Board approval and followed established ethical guidelines for online research.

#### 2.2.5 Data analysis

Descriptive statistics and reliability coefficients were first calculated to assess data quality. Latent Profile Analysis (LPA) was then conducted separately for the Korean and U. S. samples to account for potential cultural differences in value structure and response patterns. Due to sample size and analytic constraints, formal measurement invariance testing across cultural groups was not conducted. Instead, separate LPAs were performed to respect potential structural differences and minimize the risk of imposing culturally inappropriate profile solutions. Conducting group-specific analyses helps prevent the imposition of shared profiles that could obscure culturally distinct latent configurations, thereby reducing cultural bias in model estimation (Woo et al., 2018; Gillet et al., 2018). Since LPA identifies subgroups based on individual response patterns, combining culturally distinct groups may mask meaningful heterogeneity. Prior research suggests that culture-specific LPA models yield more valid interpretations of motivational structures embedded in diverse sociocultural contexts (Spurk et al., 2020; Woo et al., 2018).

Models with one to six profiles were tested using fit indices including the Akaike Information Criterion (AIC), Bayesian Information Criterion (BIC), entropy values, and likelihood-ratio tests (LMR-LRT, BLRT). Profile selection was guided by statistical fit, theoretical interpretability, and a minimum profile size of at least 5% of the sample.

Following profile identification, one-way ANOVAs tested for mean differences in intercultural sensitivity and IU across profiles. Multinomial logistic regression analyses were then conducted to determine the extent to which ISS, IU, and gender predicted profile membership. All analyses were performed using R (R Core Team, 2020), RStudio (RStudio Team, 2020), and IBM SPSS Statistics Version 28.

## 3 Results

### 3.1 Descriptive statistics and correlations

Table 3 presents descriptive statistics for all study variables. Most means exceeded 3.0, except for inhibitory anxiety. Variables generally showed negative skewness, but all distributions met univariate normality criteria (George and Mallery, 2024). Correlations among human values were positive, with humility strongly correlated with conformity (0.52) and face with several values (e.g., security, 0.58). Notably, intercultural sensitivity subscales (interaction engagement, respect for cultural differences, interaction confidence, interaction enjoyment) showed small-to-moderate negative correlations with power, conformity, and tradition (range: −0.02 to −0.36). Intolerance of uncertainty variables showed minor negative correlations with stimulation and the most intercultural sensitivity dimensions.

**Table 3:** Descriptive statistics of the research variables.

Variable	Mean	SD	Skewness	Kurtosis
Self-Direction (SD)	4.50	0.80	−0.11	−0.51
Stimulation (ST)	3.81	1.07	−0.05	−0.61
Hedonism (HE)	4.65	0.84	−0.27	−0.50
Achievement (Ach)	4.36	0.98	−0.15	−0.72
Power (PO)	3.87	0.80	0.03	−0.18
Security (SEC)	4.49	0.77	−0.12	−0.57
Conformity (CON)	4.16	0.85	−0.28	0.63
Tradition (TD)	3.12	1.06	0.19	−0.39
Humility (HM)	3.83	0.89	−0.09	−0.43
Benevolence (BEN)	4.33	0.84	−0.08	−0.46
Universalism (UNI)	4.22	0.79	0.01	−0.49
Face (FC)	4.34	0.87	0.01	−0.49
Interaction engagement (EG)	3.52	0.55	0.01	0.39
Respect for cultural difference (RD)	3.68	0.53	−0.15	−0.16
Interaction confidence (CF)	3.05	0.73	−0.14	0.23
Interaction enjoyment (EJ)	3.67	0.68	−0.36	0.38
Interaction attentiveness (AT)	3.40	0.64	−0.16	0.60
Prospective anxiety (PS)	3.31	0.70	−0.14	0.26
Inhibitory anxiety (IH)	2.81	0.89	0.16	−0.35

Table 4 presents descriptive statistics and correlations for the U. S. sample. Patterns were broadly consistent but somewhat more differentiated. In this group, interaction confidence and interaction enjoyment showed strong positive correlations with stimulation, self-direction, and universalism. Both prospective and inhibitory IU were significantly negatively correlated with intercultural sensitivity dimensions and openness-related values, while showing weak to moderate positive correlations with conservation values (e.g., security and conformity). These results suggest that intolerance of uncertainty may serve as a key psychological constraint on intercultural engagement in the U. S. sample.

**Table 4:** Descriptive statistics of the research variables.

Variable	Mean	SD	Skewness	Kurtosis
Self-Direction (SD)	4.01	0.60	−0.44	−0.33
Stimulation (ST)	3.82	0.74	−0.34	−0.18
Hedonism (HE)	4.02	0.69	−0.44	−0.41
Achievement (Ach)	3.99	0.70	−0.54	−0.34
Power (PO)	3.87	0.79	−0.20	−0.35
Security (SEC)	2.99	0.60	0.93	0.10
Conformity (CON)	3.76	0.72	−0.10	−0.67
Tradition (TD)	3.41	1.03	−0.23	−0.59
Humility (HM)	3.10	0.68	0.10	−0.98
Benevolence (BEN)	3.65	0.61	−0.62	−0.54
Universalism (UNI)	3.95	0.61	−0.24	−0.28
Face (FC)	3.53	0.77	−0.15	−0.49
Interaction engagement (EG)	3.90	0.47	−0.56	0.96
Respect for cultural difference (RD)	4.18	0.60	−0.99	1.22
Interaction confidence (CF)	3.56	0.68	−0.91	5.17
Interaction enjoyment (EJ)	4.00	0.76	−1.24	2.00
Interaction attentiveness (AT)	3.76	0.59	−0.20	0.18
Prospective anxiety (PS)	3.32	0.70	−0.90	0.22
Inhibitory anxiety (IH)	2.69	0.98	0.26	−0.55

The pattern of correlations across both samples supports the theoretical links among personal values, intercultural sensitivity, and IU, and provides preliminary justification for modeling latent value profiles and examining the psychological predictors of profile membership.

### 3.2 Latent profile analysis

The latent profile analysis (LPA) revealed distinct value configurations among undergraduate students in Korea and the United States, reflecting culturally shaped patterns of value prioritization. For the Korean sample, a five-profile solution emerged as optimal, while the U. S. sample yielded a four-profile structure. These solutions were selected based on a synthesis of statistical indices (AIC, BIC, entropy, BLRT), theoretical interpretability, and profile distribution adequacy (Ferguson et al., 2020). These distinct solutions align with expectations drawn from tightness–looseness theory (Gelfand et al., 2011) and Confucian collectivism versus Western individualism frameworks. Specifically, the Korean sample exhibited more tradition- and security-focused profiles consistent with a tight, norm-enforcing cultural context, while the U. S. profiles showed greater diversity and polarization, reflecting a loose cultural orientation and greater individualistic value prioritization.

#### 3.2.1 Korean sample

Table 5 presents the model fit indices for the one- through six-profile solutions in the Korean sample. The five-profile solution was selected as optimal, as it demonstrated the lowest AIC and BIC among viable models, high entropy (0.83), and a significant BLRT result compared to the four-profile model. The six-profile model did not yield a meaningful improvement in fit and included a class with less than 5% of the sample, which violates recommended guidelines for LPA class sizes.

**Table 5:** Latent profile analysis model fit.

Model	Log-likelihood	AIC	BIC	Entropy	LMR-LRT (p)
1-profile		17642.18	17744.13	1.00	
2-profile	−6697.98	15669.50	15826.68	0.97	916.47 (0.001)
3-profile	−6651.82	15291.86	15504.26	0.92	482.50 (0.001)
4-profile	−6551.24	15104.77	15372.40	0.91	240.91 (0.001)
5-profile	−6517.27	14883.26	15206.11	0.88	245.47 (0.001)
6-profile	−6479.21	14817.32	15195.40	0.79	92.55 (0.058)

#### 3.2.2 Standardized value profiles in Korea (five-profile solution)

Integrative Traditionalists (41%). This profile was distinguished by consistently high scores across nearly all value dimensions, particularly conservation (tradition *z* = 0.94, conformity *z* = 0.81) and self-transcendence (universalism *z* = 0.92, benevolence *z* = 0.82). Participants in this group endorsed both stability- and openness-oriented values, suggesting a comprehensive motivational framework encompassing both collectivist and individualist dimensions.

Low Tradition Endorsement (24.2%). This group showed generally below-average scores on all values. However, modestly higher scores on tradition (*z* = −0.18), humility (*z* = −0.42), and conformity (*z* = −0.41) indicate a latent preference for social avoidance or inhibition. The profile likely reflects individuals who, although not strongly endorsing any particular value orientation, retain some deference to traditional norms while exhibiting cautious self-presentation.

Change-Oriented (21.1%). This profile was defined by elevated openness-to-change values such as hedonism (*z* = 0.48) and self-direction (*z* = 0.28), combined with low endorsement of tradition (*z* = −0.53) and conformity (*z* = −0.47). Participants in this group appear to prioritize personal autonomy and experiential stimulation over societal expectations or collective norms.

Low Tradition/High Openness Profile (17.5%). Participants in this group scored above average on values associated with growth and openness (e.g., universalism *z* = 0.74, self-direction *z* = 0.49) and below average on values associated with self-protection or anxiety-avoidance (e.g., power *z* = −0.54, conformity *z* = −0.39). This profile reflects a secure, ethically oriented value structure with high intercultural flexibility.

Tradition-Oriented (21.8%). Characterized by moderate-to-high scores on security (*z* = 0.13), power (*z* = 0.10), and tradition (*z* = 0.24), this profile reflects a motivational orientation toward control, stability, and social conformity. Scores on openness values such as stimulation and self-direction were near or below average. Compared to the Growth-and-Anxiety-Free profile, this group showed a reactive stance, emphasizing social safety over proactive engagement.

**Figure 2:**
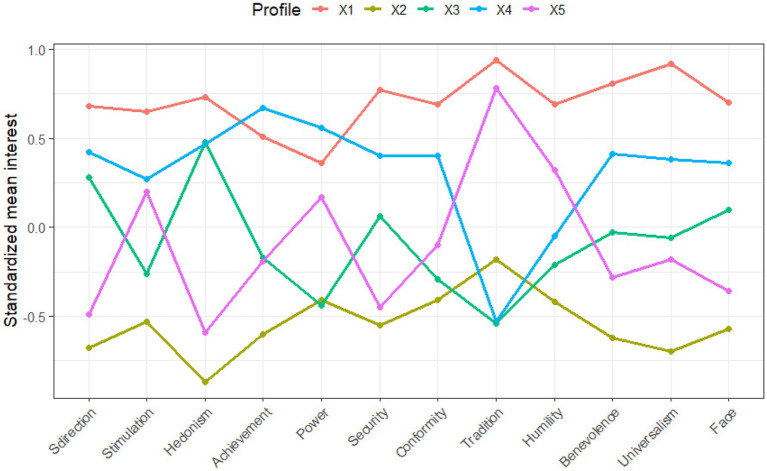
Standardized value profiles in Korea (five-profile solution).

These five profiles together represent a complex constellation of motivational value patterns within Korean undergraduates, ranging from universalist-integrated to inhibition-avoidant orientations (see Figure 2). The presence of both integrated and compartmentalized value structures highlights the shifting landscape of Korean youth identity in relation to tradition and modernity.

#### 3.2.3 United States sample

For the U. S. sample, model fit indices are also presented in Table 6. The four-profile solution was selected as optimal, balancing lower AIC and BIC values, adequate entropy (0.79), and meaningful class sizes. The five-profile solution included a class with fewer than 5% of cases and did not improve interpretability.

**Table 6:** Latent profile analysis model fit.

Model	Log-Likelihood	AIC	BIC	Entropy	LMR-LRT (p)
1-profile		14713.47	14811.07	1.00	
2-profile	−6874.727	13823.45	13973.90	0.96	916.03 (0.001)
3-profile	−6678.475	13456.95	13660.26	0.95	392.50 (0.001)
4-profile	−6566.321	13258.64	13514.81	0.94	224.30 (0.001)
5-profile	−6501.72	13155.44	13464.46	0.92	129.20 (0.001)

#### 3.2.4 Standardized means of the four-profile model

Growth-Oriented (24.8%). This profile was marked by above-average endorsement of openness and self-transcendence values, especially universalism (*z* = 0.74) and self-direction (*z* = 0.49), while values associated with self-protection and anxiety-avoidance (e.g., power *z* = −0.54, face *z* = −0.39, conformity *z* = −0.39) were rated below average. This group reflects a secure and outward-oriented value system prioritizing ethical concern and autonomy, with relatively low concern for status or social conformity.

Broad Value Endorsement (21.8%). Participants in this profile endorsed high levels on nearly all values, with z-scores exceeding 0.50 across the board. Notably, this group scored exceptionally high on face (*z* = 0.99), security (*z* = 1.00), self-direction (*z* = 0.83), and benevolence (*z* = 0.87), indicating an integrative value orientation that spans all four of Schwartz’s higher-order dimensions. This profile reflects individuals with broad motivational investment across both personal and collective domains.

Security-Focused (35.0%). This was the largest profile and showed moderately elevated scores on values such as security (*z* = 0.13), tradition (*z* = 0.24), conformity (*z* = 0.07), and power (*z* = 0.10). In contrast, openness values such as stimulation (*z* = −0.32) and self-direction (*z* = −0.18) were lower. This profile represents a more defensive and stability-seeking orientation, likely shaped by a desire for control and predictability.

Tradition-Hesitant (18.3%). Members of this group reported below-average scores across all value domains, with particularly low scores on self-direction (*z* = −1.33), achievement (*z* = −1.25), and benevolence (*z* = −1.23). Tradition (*z* = −0.19) and power (*z* = −0.22) were closest to average. This pattern suggests a disengaged or uncertain motivational stance, potentially reflecting ambivalence toward both traditional and progressive value domains.

**Figure 3:**
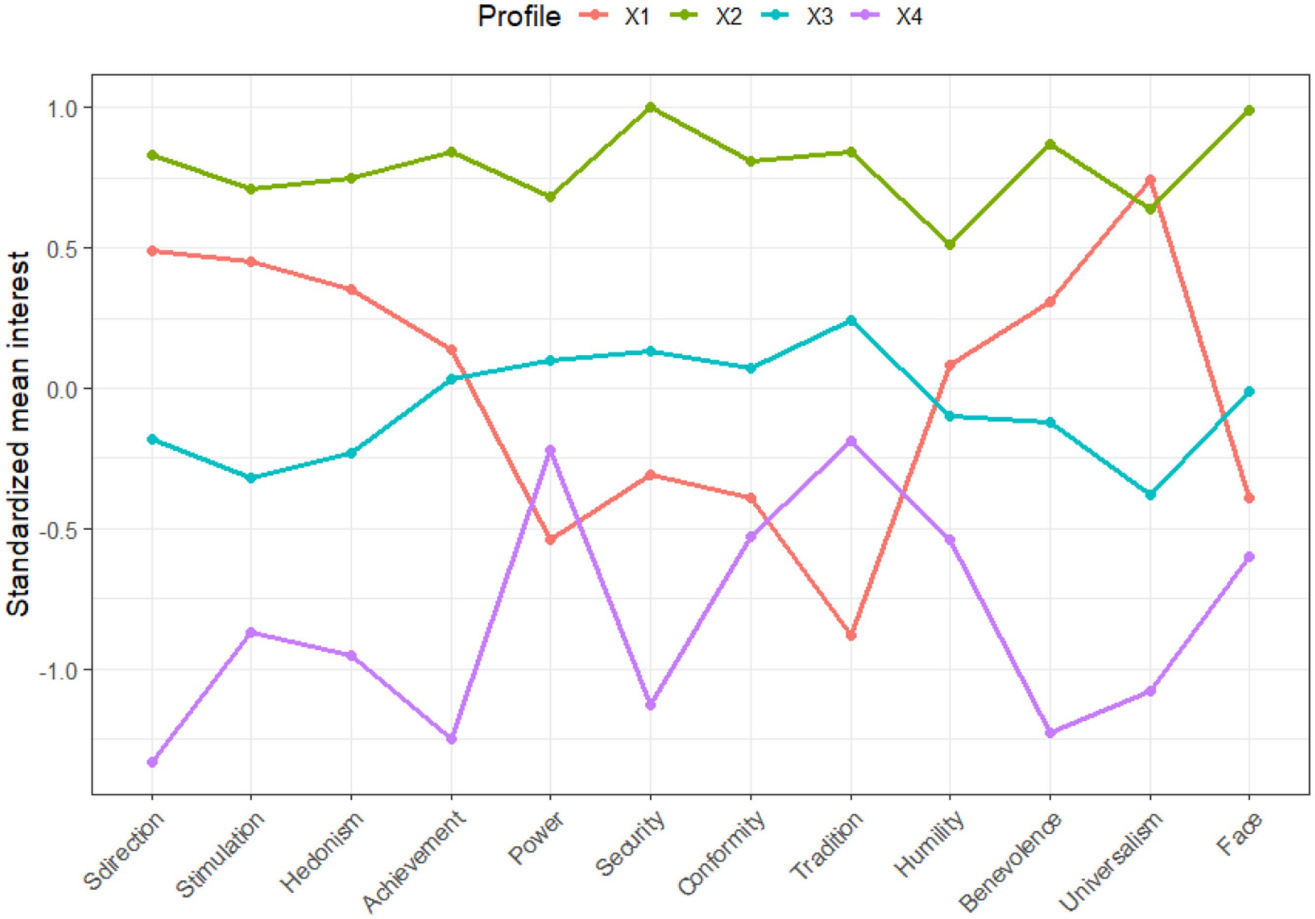
Standardized means of the four-profile model. Within-profile standard deviations are not provided because the model specified equal variance constraints across classes for estimation stability. Readers should interpret mean differences as indicative of dominant patterns, but caution is warranted given this limitation.

Together, these four profiles highlight the diversity of value structures among American undergraduates (see Figure 3). The profiles ranged from expansive value integration (All Value Endorsed) to selective endorsement (Self-Protection), and even disengagement (Low Tradition Endorsement), suggesting meaningful subcultural variation within a single national sample.

Naming conventions were determined by examining the dominant value dimensions within each profile, aligned with Schwartz’s value theory (2012). All labels were phrased to minimize evaluative connotations and to reflect relative patterns rather than fixed traits. The Appendix Table 1A summarizes the defining value characteristics and rationale for each profile name across both cultural groups.

### 3.3 Covariate/predictor analyses

Multinomial logistic regression analyses examined how intercultural sensitivity (ISS), intolerance of uncertainty (IU), and gender predicted membership in the derived value profiles. For each country, the most representative profile served as the reference group. Odds ratios (ORs), 95% confidence intervals (CIs), and exact *p*-values are reported. All models controlled for age and academic year.

#### 3.3.1 Korean sample

Multinomial logistic regression analyses revealed distinct psychological and intercultural predictors of membership in the derived value profiles, relative to the Integrated Values reference group (Table 7). For the Low Tradition Endorsement Profile, a one-point increase in interaction engagement was associated with a 52% reduction in membership odds (OR = 0.48, 95% CI [0.31, 0.75], *p* = 0.001), while greater respect for cultural differences similarly decreased the odds by 42% (OR = 0.58, 95% CI [0.40, 0.85], *p* = 0.005). These findings align with Schwartz’s conservation dimension, suggesting that ambivalence toward intercultural engagement reinforces a cautious adherence to traditional norms.

**Table 7:** Multinomial logistic regression result.

Predictor	*b*	SE	OR	95% CI	*p*-value
Low tradition endorsement					
Interaction engagement	−0.74	0.22	0.48	[0.31, 0.75]	0.001
Respect for differences	−0.54	0.18	0.58	[0.40, 0.85]	0.005
Change-oriented					
Interaction confidence	−0.8	0.24	0.45	[0.28, 0.72]	0.001
Interaction enjoyment	0.52	0.21	1.68	[1.12, 2.53]	0.012
Tradition-oriented					
Gender (female)	0.76	0.27	2.14	[1.24, 3.69]	0.006
Interaction Engagement	−0.94	0.23	0.39	[0.25, 0.61]	<0.001
Prospective IU	−0.6	0.22	0.55	[0.35, 0.86]	0.009

The Change-Oriented Profile exhibited a dual dynamic: lower interaction confidence predicted membership (OR = 0.45, 95% CI [0.28, 0.72], *p* = 0.001), whereas higher interaction enjoyment increased the odds by 68% (OR = 1.68, 95% CI [1.12, 2.53], *p* = 0.012). This tension reflects the interplay between anxiety-avoidance (diminished confidence in intercultural interactions) and openness to change (heightened enjoyment of cultural diversity), central to Schwartz’s model of value conflicts.

Membership in the Tradition-Emphasized Profile was strongly gendered, with female students showing 2.14 times higher odds than males (95% CI [1.24, 3.69], *p* = 0.006). Concurrently, interaction engagement reduced the odds by 61% (OR = 0.39, 95% CI [0.25, 0.61], *p* < 0.001), underscoring the role of Confucian gender norms in perpetuating tradition-centric value hierarchies.

The model demonstrated robust explanatory power [Nagelkerke pseudo *R*^2^ = 0.22, *χ*^2^(24) = 167.32, *p* < 0.001], confirming the salience of intercultural sensitivity and intolerance of uncertainty in shaping value profiles within Korea’s collectivist context. These findings support Hypothesis 2-that individual differences in intercultural competence and uncertainty tolerance differentiate value profile membership. Specifically, profiles characterized by lower openness to change (e.g., Tradition-Emphasized) or weaker integration of tradition with growth-oriented values (e.g., Low Tradition Endorsement) exhibited reduced intercultural confidence and stronger inhibitory tendencies (e.g., avoidance of ambiguity). This pattern underscores how psychological dispositions (e.g., uncertainty intolerance) and behavioral tendencies (e.g., intercultural avoidance) interact to reinforce distinct value hierarchies in Confucian-influenced settings.

#### 3.3.2 United States sample

Multinomial logistic regression analyses identified distinct predictors of value profile membership, with the Integrated Values profile (characterized by uniformly high endorsement across all values) serving as the reference group (Table 8). This profile was selected as the baseline for comparison due to its theoretical neutrality, allowing clear interpretation of deviations toward anxiety-avoidance or growth-oriented orientations within Schwartz’s framework of self-enhancement versus self-transcendence.

**Table 8:** Multinomial logistic regression result.

Predictor	*b*	SE	OR	95% CI	*p*-value
Growth-oriented					
Interaction confidence	−0.51	0.19	0.6	[0.41, 0.88]	0.009
Security-focused					
Interaction engagement	−0.71	0.2	0.49	[0.33, 0.73]	<0.001
Interaction confidence	−0.4	0.18	0.67	[0.47, 0.96]	0.028
Low tradition endorsement					
Interaction engagement	−1.1	0.25	0.33	[0.20, 0.55]	<0.001
Prospective IU	−0.69	0.24	0.5	[0.31, 0.81]	0.005

For the Security-Focused profile, a one-point increase in interaction engagement reduced membership odds by 51% (OR = 0.49, 95% CI [0.33, 0.73], *p* < 0.001), while lower interaction confidence (OR = 0.67, 95% CI [0.47, 0.96], *p* = 0.028) and attentiveness (OR = 0.70, 95% CI [0.51, 0.96], *p* = 0.027) further reinforced anxiety-avoidance tendencies. These findings align with Schwartz’s self-protection dimension, where prioritizing stability and risk mitigation overrides openness to intercultural experiences.

For the Low Tradition Endorsement profile, diminished interaction engagement (OR = 0.33, 95% CI [0.20, 0.55], *p* < 0.001) and lower prospective intolerance of uncertainty (OR = 0.50, 95% CI [0.31, 0.81], *p* = 0.005) predicted membership. This reflects the interplay between uncertainty intolerance and value ambivalence in individualistic contexts, where hesitancy to commit to cultural norms coexists with fragmented adherence to tradition.

For the Growth-Focused profile, lower interaction confidence reduced membership odds by 40% (OR = 0.60, 95% CI [0.41, 0.88], *p* = 0.009), underscoring the role of intercultural assurance in fostering Schwartz’s growth-anxiety framework. This profile epitomizes the tension between self-transcendent aspirations (e.g., universalism) and the psychological barriers imposed by intercultural uncertainty.

The model accounted for 18% of variance in profile membership [Nagelkerke pseudo *R*^2^ = 0.18, *χ*^2^(18) = 132.45, *p* < 0.001], underscoring the moderate yet significant role of intercultural sensitivity and uncertainty management in shaping value hierarchies within the U. S. sample. These results align with Schwartz’s assertion that individualistic societies prioritize dynamic trade-offs between self-enhancement (e.g., achievement, power) and communal well-being (e.g., universalism, benevolence), mediated by psychological dispositions toward risk and ambiguity.

Supporting Hypothesis 2, the findings reveal that motivational diversity in individualistic contexts arises from measurable differences in intercultural orientation and uncertainty tolerance. Specifically, profiles emphasizing ethical concern and openness (e.g., Growth-Focused) were distinguished by higher interaction confidence and engagement, whereas those prioritizing psychological security (e.g., Security-Seeking) exhibited inhibitory tendencies and aversion to ambiguity. This bifurcation reinforces the centrality of intercultural competence as both a driver and marker of value integration in pluralistic societies, where proactive engagement with diversity aligns with Schwartz’s growth-anxiety framework.

In sum, the latent profile analysis identified both shared and culturally distinct value configurations among Korean and U. S. undergraduates, with profile membership systematically predicted by intercultural sensitivity, intolerance of uncertainty, and, in the Korean sample, gender. Detailed interpretation and theoretical implications of these findings are addressed in the Discussion.

## 4 Discussion

This study provides a novel empirical framework for understanding how individual-level motivational systems are structured within broader cultural contexts, shaped by intercultural sensitivity (ISS) and intolerance of uncertainty (IU). By employing latent profile analysis (LPA), we uncovered distinct value configurations among Korean and U. S. undergraduates that reflect cultural traditions, psychological readiness to engage with diversity, and culturally conditioned responses to ambiguity. Our findings extend Schwartz’s value theory and intercultural competence research by demonstrating how cultural tightness and uncertainty regulation differentially sculpt value integration and conflict management strategies.

### 4.1 Culturally embedded motivational configurations

In both countries, we found profiles along Schwartz’s openness-conservation continuum, but with revealing structural differences. In the Korean sample, Integrative Traditionalists (41%) combined tradition, universalism, and openness—demonstrating how collectivist societies can reconcile Confucian heritage with globalized ethics. This challenges the assumed opposition between conservation and openness in Schwartz’s model and aligns with recent work on cultural tightness (Gelfand et al., 2006), where strong norms coexist with adaptive hybridization, which reflects how individuals negotiate competing value demands within a tight cultural context. This is consistent with Schwartz’s (2012) assertion that value structures can be fluid, with openness and conservation goals coexisting in response to contextual constraints. The Low Tradition/High Openness Profile (17.5%), characterized by high universalism but rejection of conformity, mirrors Syrtsova’s (2014) findings that uncertainty intolerance can manifest as ethical non-conformity in cultures with high uncertainty avoidance.

The U. S. sample, however, showed polarization into Growth-Oriented (24.8%) and Security-Focused (35.0%) profiles, reflecting individualistic “looseness” where anxiety avoidance drives compartmentalization rather than integration. The Low Tradition Endorsement group (18.3%), showing broad value disengagement, corresponds with research on “weak uncertainty avoidance” cultures (Taras et al., 2016), where fragmented identities emerge from unregulated pluralism. This division highlights that in individualistic contexts, intolerance of uncertainty functions as a personal resilience deficit, whereas in collectivist Korea, it reinforces socially scripted conservatism.

### 4.2 Psychological predictors: ISS, IU, and culturally conditioned anxiety

Our results validate IU as a cross-cultural predictor of value compartmentalization, yet its mechanisms diverge. In Korea, inhibitory IU amplified tradition adherence (OR = 0.55, *p* = 0.009), particularly among women, reflecting Confucian gender norms that prescribe risk aversion and relational harmony. The gender effect emerging exclusively in the Korean sample likely reflects the persistent influence of Confucian gender expectations. Within traditional Korean society, women have historically been guided by the principle of ‘three obediences,’ which emphasized filial devotion and family loyalty, cultivating orientations toward social harmony, traditional values, and collective stability (Ren et al., 2024). Cross-cultural research across China, Vietnam, and Japan demonstrates that women in Confucian-influenced societies continue to internalize these cultural expectations, particularly in contexts where traditional frameworks remain salient (Gao et al., 2012; Grosse, 2015). Ren et al. (2024) observed that while both male and female Chinese youth strongly valued filial obligations, nuanced gender differences emerged in their endorsement of humility and face-saving behaviors, indicating that female students remain more deeply socialized toward family-centered value systems. Similarly, research in Hanoi revealed that young Vietnamese women expressed nearly equivalent agreement with traditional gender-role expectations as their male counterparts, reflecting the continued strength of Confucian patriarchal structures (Gao et al., 2012). These findings suggest that Confucian cultural frameworks may intensify women’s propensity toward tradition-oriented and security-focused motivational configurations. This aligns with Liu and Almor’s (2016) assertion that tight cultures encode uncertainty intolerance into social roles. Conversely, U. S. profiles linked prospective IU to security-seeking (OR = 0.50, *p* = 0.005), where anxiety about unpredictable outcomes-not social expectations-drives defensive values, consistent with Lee et al.’s (2024) findings on IU’s role in maladaptive emotion regulation.

The dual role of intercultural sensitivity further highlights cultural differences: Korean Change-Oriented students showed high enjoyment but low confidence, a tension reflecting Buhr and Dugas (2002) “anxiety-ambition paradox” in Confucian-influenced individualism. In the U. S., interaction confidence proved crucial for growth profiles (OR = 0.60, *p* = 0.009), suggesting that ethical idealism requires psychological assurance in loose cultures. These findings reposition intercultural sensitivity and uncertainty intolerance not simply as traits but as identity-regulating mechanisms that govern the integration of ethical ideals within cultural frameworks.

### 4.3 Theoretical advancements

This study advances value theory by challenging the presumed incompatibility of conservation and openness values in Schwartz’s original model. The Korean Integrative Traditionalists profile-characterized by the coexistence of tradition (*z* = 0.94), universalism (*z* = 0.92), and self-direction (*z* = 0.28)-demonstrates that cultural tightness (Gelfand et al., 2011) can foster adaptive hybridization rather than polarization. This finding aligns with Schwartz’s (2006) revised cultural value model, where societal norms modulate value integration, enabling collectivist societies to reconcile Confucian heritage with globalized ethics. By contrast, the U. S. sample’s polarization into Growth-Oriented and Security-Focused profiles reflects the individualistic imperative to resolve anxiety through discrete self-enhancement or self-protection strategies, reinforcing the conservation-openness dichotomy in loose cultures.

The role of intolerance of uncertainty (IU) as a cultural scaffold further clarifies how anxiety regulation shapes value systems. In Korea’s tight cultural context, inhibitory IU reinforced gendered conservatism (OR = 0.55, *p* = 0.009), encoding uncertainty avoidance into socially prescribed roles. Conversely, in the U. S., prospective IU exacerbated polarization, driving security-seeking behaviors (OR = 0.50, *p* = 0.005) through personal anxiety rather than collective norms. This dual function of IU supports Gelfand et al.’s (2011) assertion that cultural tightness determines whether uncertainty avoidance manifests as social conformity or individualized anxiety.

Finally, intercultural sensitivity (ISS), particularly interaction confidence, emerged as a resilience marker against value fragmentation. In both samples, higher ISS predicted membership in growth-oriented profiles (Korea: OR = 1.68, *p* = 0.012; U. S.: OR = 0.60, *p* = 0.009), extending Chen and Starosta’s (2000) model by positioning ISS as a buffer in pluralistic environments. This suggests that ISS may function not merely as a communicative skill but also as a psychological resource that can help support a sense of ethical coherence in an era of global uncertainty. However, this interpretation should be treated with caution given the cross-sectional nature of the data.

### 4.4 Practical implications

The identified value profiles and their predictors offer actionable insights for educators, institutions, and mental health practitioners. In Korea, tradition-oriented students exhibiting inhibitory IU may benefit from pedagogical strategies that reframe ambiguity as an opportunity for ethical growth. Case-based learning on dilemmas like balancing filial piety with gender equality could help students navigate conflicting values while preserving cultural identity.

In U. S. contexts, security-focused students hindered by prospective IU require interventions that build intercultural problem-solving skills. Experiential training-such as simulated cross-cultural negotiations or collaborative projects with international peers-could reduce anxiety by fostering concrete competencies in ambiguity management. Globally, screening high-IU students for value-behavior misalignment-a predictor of anxiety disorders (Shu et al., 2022)-could enable early interventions. Universities might integrate value clarification exercises into orientation programs, helping students align their motivations with academic and career choices.

### 4.5 Limitations and directions for future research

This study has several limitations. The cross-sectional design restricts causal inference; longitudinal research is needed to examine how value profiles evolve over time and in response to life transitions or macro-level social events. Reliance on self-report measures introduces potential bias, particularly in collectivist cultures where social desirability pressures are stronger. Our focus on university students also limits generalizability, and although separate LPAs were conducted to account for cross-cultural non-invariance, future work should explore formal measurement equivalence. The absence of formal measurement invariance testing means that direct comparisons of profile structures across cultures should be interpreted with caution. Future studies should test measurement equivalence with larger, more diverse samples to strengthen the validity of cross-cultural interpretations. This is especially salient in collectivist contexts like South Korea, where strong norm-conformity pressures and the identity exploration typical of emerging adulthood may shape value expression in ways that differ from other age groups or community samples. While our person-centered, cross-sectional LPA approach did not formally test these pathways, future studies should employ mediation and moderation analysis to clarify whether IU serves as a mediator between personal values and ISS, and whether cultural context moderates these pathways. Longitudinal or experimental designs will be especially valuable for examining how these mechanisms unfold over time.

Building on these findings, several directions for future research are warranted. First, longitudinal and experimental designs are needed to clarify the causal pathways linking value orientation, intercultural sensitivity, and intolerance of uncertainty—especially as individuals navigate major developmental transitions such as emerging adulthood, workforce entry, or migration. Such designs would allow researchers to examine how motivational structures evolve over time and in response to life events or changing sociocultural environments. Moreover, the observed cross-cultural differences in value profiles are broadly consistent with tightness–looseness theory and Confucian versus Western individualism frameworks (Gelfand et al., 2011; Markus and Kitayama, 1991). Future research should continue to examine how these cultural contexts condition motivational configurations and the role of IU in different societies. In addition, future studies should recruit more diverse age groups and non-student samples to assess whether these profiles hold across broader segments of the population.

Second, there is a clear need to replicate and extend this study in non-student populations and across underrepresented cultural contexts. Future research should include working adults, adolescents, and participants from African, Middle Eastern, and Latin American societies, where value systems and identity development may follow distinct trajectories shaped by different religious, historical, and institutional structures.

Third, qualitative and narrative approaches would deepen understanding of how individuals subjectively experience and negotiate value tensions in daily life. These methods could illuminate the lived experiences behind profiles such as Integrative Traditionalists or Low Tradition/High Openness Profile youth, offering richer insight into how hybrid motivational systems are embodied and maintained.

Fourth, future studies should explore the influence of digital media, transnational networks, and global youth culture on value integration and fragmentation. As digital technologies increasingly mediate intercultural exposure and identity construction, understanding their role in shaping motivational coherence is essential.

Finally, researchers should design and test intervention programs that aim to enhance intercultural sensitivity and uncertainty tolerance, particularly among individuals in hesitant or risk-averse profiles. Evaluating whether such interventions can promote shifts in value profile membership—and whether these shifts correspond to improvements in psychological well-being, identity coherence, or social adaptability—will provide critical applied knowledge for educators, counselors, and organizational leaders.

## Data Availability

The datasets generated and analyzed in this study are not publicly available due to IRB restrictions and participant confidentiality. Data sharing is not permitted under the terms of the approved research protocol. Requests to access the datasets should be directed to Jungsun Kim, kim929@purdue.edu.

## Appendix

**Table A1:** Profile naming.

Profile name	Defining value pattern	Naming rationale	Notes on interpretation
Korea sample			
Broad value endorsement	High across all value domains	Reflects general high endorsement of all Schwartz dimensions	May reflect acquiescence bias
Low tradition endorsement	Low on tradition/conformity, moderate on others	Indicates lower prioritization of conservation values	Not motivational disengagement
Low tradition/high openness profile	Low conservation, high openness/universalism	Highlights preference for autonomy and change over tradition	Relative pattern only
Tradition-oriented	High on tradition, security, conformity	Emphasis on conservation and norm maintenance	Linked to tight cultural norms
Integrative traditionalists	High on tradition, universalism, moderate openness	Combines traditional and openness/self-transcendence values	Pattern integrates seemingly opposing values
U. S. sample			
Broad value endorsement	High across all value domains	Generalized high endorsement, similar to Korea sample	Possible acquiescence bias
Low tradition endorsement	Low conservation, average others	Low priority for tradition and security	May indicate motivational diffusion
Change-oriented	High openness/universalism, low tradition/conformity	Highlights emphasis on self-direction and growth-oriented values	Pattern emphasizes autonomy over conservation
Tradition-oriented	High on tradition, security, conformity	Strong conservation orientation	Consistent with tight norm maintenance
